# Discovery of Novel Viruses in Mosquitoes from the Zambezi Valley of Mozambique

**DOI:** 10.1371/journal.pone.0162751

**Published:** 2016-09-28

**Authors:** Harindranath Cholleti, Juliette Hayer, Ana Paula Abilio, Fernando Chanisso Mulandane, Jenny Verner-Carlsson, Kerstin I. Falk, Jose M. Fafetine, Mikael Berg, Anne-Lie Blomström

**Affiliations:** 1 Section of Virology, Department of Biomedical and Veterinary Public Health, Swedish University of Agricultural Sciences (SLU), Uppsala, Sweden; 2 SLU Global Bioinformatics Centre, Department of Animal Breeding and Genetics, Swedish University of Agricultural Sciences, Uppsala, Sweden; 3 National Institute of Health, Maputo, Mozambique; 4 Division of Molecular Diagnostics and Epidemiology, Biotechnology Center, Eduardo Mondlane University, Maputo, Mozambique; 5 Public Health Agency of Sweden, Solna, Sweden; 6 Department of Microbiology, Tumor and Cell Biology (MTC), Karolinska Institute, Stockholm, Sweden; Defence Research Laboratory, INDIA

## Abstract

Mosquitoes carry a wide variety of viruses that can cause vector-borne infectious diseases and affect both human and veterinary public health. Although Mozambique can be considered a hot spot for emerging infectious diseases due to factors such as a rich vector population and a close vector/human/wildlife interface, the viral flora in mosquitoes have not previously been investigated. In this study, viral metagenomics was employed to analyze the viral communities in *Culex* and *Mansonia* mosquitoes in the Zambezia province of Mozambique. Among the 1.7 and 2.6 million sequences produced from the *Culex* and *Mansonia* samples, respectively, 3269 and 983 reads were classified as viral sequences. Viruses belonging to the *Flaviviridae*, *Rhabdoviridae* and *Iflaviridae* families were detected, and different unclassified single- and double-stranded RNA viruses were also identified. A near complete genome of a flavivirus, tentatively named Cuacua virus, was obtained from the *Mansonia* mosquitoes. Phylogenetic analysis of this flavivirus, using the NS5 amino acid sequence, showed that it grouped with ‘insect-specific’ viruses and was most closely related to Nakiwogo virus previously identified in Uganda. Both mosquito genera had viral sequences related to Rhabdoviruses, and these were most closely related to *Culex tritaeniorhynchus rhabdovirus* (CTRV). The results from this study suggest that several viruses specific for insects belonging to, for example, the *Flaviviridae* and *Rhabdoviridae* families, as well as a number of unclassified RNA viruses, are present in mosquitoes in Mozambique.

## Introduction

Mosquitoes are important arboviral vectors with the potential to carry and spread pathogenic viruses to both humans and animals. It has been estimated that half of the global population is at risk of mosquito-borne viral infections and that these types of infections cause millions of deaths each year. Yellow fever virus, dengue virus, chikungunya virus, Japanese encephalitis virus and Rift Valley fever virus are examples of mosquito-borne viruses responsible for disease outbreaks in human and animal populations [1]. Apart from known pathogenic viruses it has been shown that arthropods harbor a diverse range of viruses with the potential capability of infecting humans, animals and plants [2–4]. In addition to vertebrate and plant viruses, viruses specific to arthropods, known as insect-specific viruses (ISVs), have also been identified in different mosquito populations across the world. These viruses are distantly related to known pathogenic viruses of different viral families such as *Flavivirdae*, *Bunyaviridae*, *Rhabdoviridae*, *Togavridae* and *Reoviridae* [5–7].

The virus discovery rate in different species, such as mosquitoes, have increased through the availability of deep-sequencing technologies combined with viral metagenomic approaches. Apart from novel virus discovery, viral metagenomics have become a powerful method for genetically characterizing the complete viral populations of different sample [2, 3, 8, 9]. The aim of the current study was to use viral metagenomics, combined with deep-sequencing, to identify mosquito-borne viruses circulating in the Zambezia province in Mozambique. The Zambezia province was chosen in this study as it can be considered a so-called hot spot, where new emerging infectious diseases (EIDs) are most likely to originate. This because the region is not only rich in arthropod vectors and wildlife species but there is also a close proximity between wildlife, domestic animals and humans, making viral transmission from one species to another more likely to occur, which is one of the predictors for originating new EIDs [10].

## Methods

### Sample collection and taxonomic identification

A total of 540 mosquitoes were collected in Cuacua (co-ordinates- S17°48.043' E 035°24.730), Zambezia province in Mozambique from October—November 2014 using CDC light traps. Individual mosquitoes were identified morphologically to determine the genus according to the descriptions of Edwards FW, 1941 and Hopkins GHE, 1952 [11, 12] and stored in RNAlater (Ambion) until further use. In the further analysis steps the mosquitoes belonging to the two genera were handled separately. Samples were collected on a private land with the permission from owner of the land and local formers. No specific permissions were required for these locations/activities as the study did not involve endangered or protected species.

### Nucleic acid extraction

Each mosquito pool (up to 20 mosquitoes/pool) was mechanically homogenized using the Tissuelyser II (Qiagen) for 30 cycles/sec with 1 ml of TRIzol LS reagent (Invitrogen) and two 5 mm stainless steel beads. A centrifugation step (13000 g for 10 min) was performed, and the supernatant was collected for nucleic acid extraction. Total RNA was extracted from the TRIzol/mosquito homogenate according to the manufacturer’s instructions (Life Technologies), and the RNA pellet was dissolved in 40 μl of nuclease-free water. Five microliters of RNA from each pool was pooled, and the RNA was then concentrated and subjected to DNase treatment using RNeasy MinElute Cleanup kit (Qiagen) and RNAse-free DNase set (Qiagen). Ribosomal RNA was removed using a RiboZero kit (Ribo-Zero Gold (Human/Mouse/Rat), Illumina) according to the manufacturer’s protocol, and the RNA was again concentrated using RNeasy MinElute Cleanup kit (Qiagen).

### cDNA library preparation, amplification and sequencing

Ten microliters of RNA were used in the cDNA synthesis step, which also labelled the cDNA at the both ends with a tag-sequence. The first-strand synthesis was performed using the primer FR26RV-N (5’GCCGGAGCTCTGCAGATATCNNNNNN’3) [13] and SuperScript III (Invitrogen) according to the manufacturer’s instructions. After the first-strand synthesis, Superscript was inactivated at 70°C for 10 min, and the sample was placed on ice for 2 min. The second-strand synthesis was performed with the addition of Klenow fragments (3’ to 5’ exo-) (New England BioLabs Inc.) to the first-strand cDNA, and the reaction was incubated for 1 h at 37°C and terminated at 75°C for 10 min. Random amplification was performed as follows: 1x PCR buffer, 2.5 mM MgCl_2_, 2.5 mM deoxynucleoside triphosphates (dNTP), 0.8 mM FR20RV (5’GCCGGAGCTCTGCAGATATC’3) and 1.25 U AmpliTaq Gold DNA polymerase (Applied Biosystems). The PCR was performed with 10 min at 95°C; followed by 40 cycles of 30 sec at 95°C, 30 sec at 58°C, and 90 sec at 72°C; and a final elongation step at 72°C for 10 min. The tag-sequence was cleaved off with EcoRV (NEB), and the product was purified using a GeneJet PCR purification kit (Thermo Fisher Scientific).

The library preparation and sequencing of the amplified PCR product was performed at the National Genomics Centre, SciLifeLab, Uppsala, Sweden. An ion-torrent PGM sequencing platform was used with a Ion 318^TM^ Chip v2 and 400 bp read length chemistry.

### Bioinformatics

Raw data were filtered to remove sequences with low quality scores (Q<20) and exact duplicate reads. The ends of the reads with low quality were trimmed using PRINSEQ [14]. The host genome was removed by mapping to the *Anopheles*, *Aedes* and *Culex* genomes with Bowtie2 [15]. Unmapped reads were extracted and then subjected to a basic local alignment search tool (BLASTn and BLASTx) querying the NCBI nucleotide (nt) and protein sequence database (nr) using an E-value cut-off of 1e-03. The identified viral reads were assembled by *de novo* assemblers using CodonCode Aligner 6.0.2 (CodonCode Corporation) and SeqMan NGen 11.2.1 (DNASTAR) to generate longer sequences. Alignment of contigs and unassembled reads to viral reference genomes was performed using CodonCode Aligner 6.0.2 (CodonCode Corporation).

### Confirmation and retrieval of near full-length viral genome sequences

Viral contigs and single reads were used to manually design specific primers to confirm the presence of the virus as well as to amplify longer genome regions of the selected viruses from the original material. Thermal cycling was initiated with a denaturation step at 95°C for 10 min; followed by 35 cycles of 95°C for 30 sec, 58–60°C 30 sec and 72°C for 1 min; and a final extension at 72°C for 7 min. All the primers used are presented in S1 Table. The PCR products were purified with a GeneJet PCR purification kit (Thermo Fisher Scientific) and sequenced at Macrogen Europe (Macrogen Inc.).

### Phylogenetic analysis

To determine the phylogenetic relationship of the selected viruses from the mosquitoes, reference viral amino acid sequences representing flaviviruses and rhabdoviruses were obtained from GenBank. Sequence alignments were performed using ClustalW, and phylogenies were generated using the Neighbor-Joining method with MEGA 6.06 software [16, 17]. The statistical significance of the tree topologies was evaluated by 1000 bootstrap replicates.

## Results

**Fig 1:**
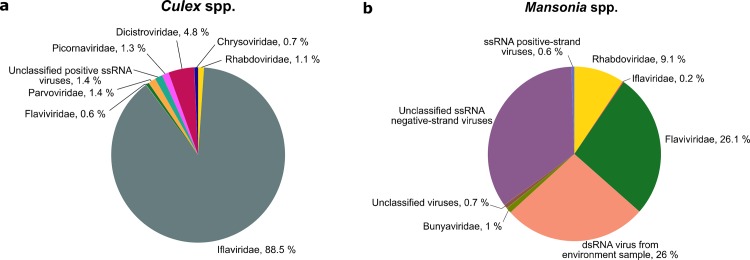
Overview of viral sequence classification of the metagenomic sequences from *Culex* spp. (a) and *Mansonia* spp. (b) at a family level.

The mosquitoes were collected in the Zambezi Valley of Zambezia province, Mozambique and were classified as *Culex* (156) and *Mansonia* (384) genera. A total of 1,733,788 and 2,623,031 sequences were obtained from the *Culex* and *Mansonia* pools, respectively (Table 1). After quality control, 2.85 and 3.6% of the reads were removed, respectively, and the remaining reads had an average length of 261 and 270 nucleotides. Twenty-one and eight percent of the sequences from *Culex* and *Mansonia* spp, respectively, mapped to the mosquito (*Anopheles*, *Aedes* and *Culex)* reference genomes. The majority of the remaining reads were classified as eukaryotic genome, mainly the host genome and as bacteria (Wolbachia, Salmonella, Acenetobacter etc). Only 0.19% (3269 reads) and 0.03% of the reads (983 reads) were classified as viral sequences in the *Culex* and *Mansonia* mosquito pools. Viral sequences from *Flaviviridae*, *Rhabdoviridae*, *Iflaviridae* were identified; in addition, unclassified single-stranded and double-stranded viral RNA sequences were also detected (Fig 1A and 1B).

**Table 1:** Basic information of the sequence data, quality filtering, host mapping and BLAST.

Sample	Total no. of reads	No. of reads after quality check	High-quality reads	Average length of high-quality reads (nt)	Reads mapped to the host genome	No. of viral reads based on BLASTx search	Percentage of viral reads
*Culex* spp.	1733788	1684319	97.15%	261.25	21.36%	3269	0.19
*Mansonia* spp.	2623031	2528583	96.4%	270.40	7.98%	983	0.03

### Flaviviruses *(Flaviviridae* family)

**Fig 2:**
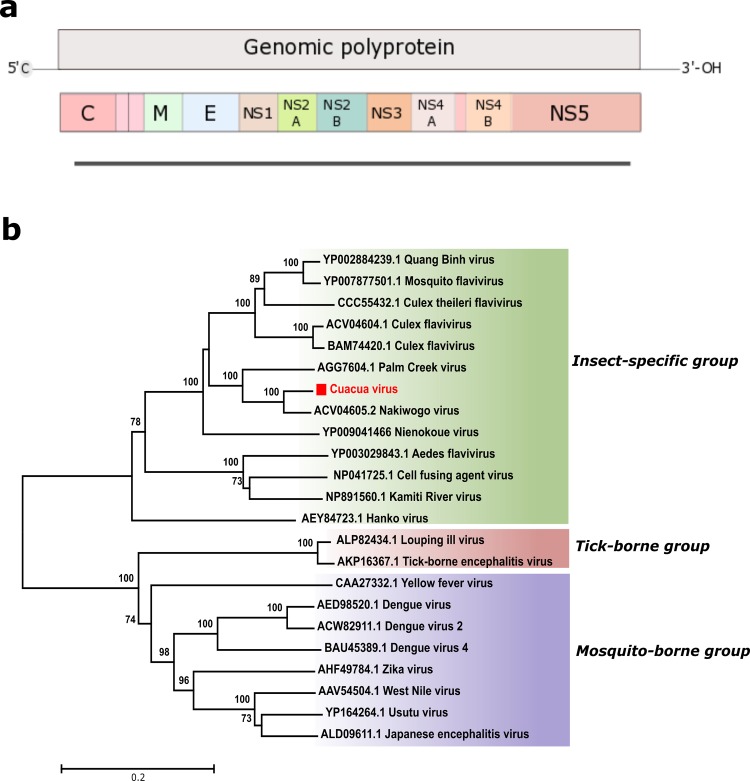
Schematic representation of the identified CuCuV genome (9747 nt) (a) and phylogenetic analysis of the selected sequence from CuCuV based on a 759 amino acid sequence of the NS5 protein (b).

In the current study, both the investigated *Culex* spp. and the *Mansonia* spp. mosquitoes had sequences related to the *Flaviviridae* family. A total of 19 reads in *Culex* spp. and 257 reads in *Mansonia* spp. were classified as flaviviral reads, constituting 1% and 26.9% of all viral reads, respectively. In the *Culex* spp., the detected flavivirus reads were most closely similar to Palm Creek virus (PCV) (GenBank KC505248.1) and Nakiwogo virus (NAKV) (GenBank GQ165809). In *Mansonia* spp., 175 reads showed highest similarity, at an amino acid level, to NAKV, which is a ssRNA positive-strand virus previously detected in *Mansonia* spp. in Uganda [18]. The reads were further assembled into 10 contigs (283–2650 nt in length). The NAKV genome was used as a reference genome, and mapping of the assembled contigs and unassembled reads resulted in an alignment covering 70% of the genome with high sequence coverage, especially in the conserved NS5 region. To sequence the full-length genome of the identified virus, PCR primers were designed, and conventional PCRs were used to fill the gaps between the contigs. Except for the 5’ and 3’ ends (150 nt each side approximately), a near full-length genome of 9747 nt was obtained (Fig 2A). Sequence comparison of this nearly complete genome showed 75 and 86% sequence identity to NAKV at the nucleotide and amino acid levels, respectively. As seen in all known insect-specific flaviviruses (ISFs), a heptanucleotide sequence required for the predicted -1 ribosomal frameshifting (-1 PRF) was also identified in the detected virus [19]. Neighbor joining was used to construct a phylogenetic tree using the conserved NS5 amino acid sequence of the identified flavivirus and other flaviviruses, including viruses from the insect-only, tick-borne and mosquito-borne groups. The identified viral sequence clustered with the ISFs and was confirmed to be most closely related to NAKV from Uganda (Fig 2B). The phylogeny of the near full-length viral polyprotein showed a similar topological arrangement as the NS5 sequence (not shown). The 15% difference from other known flaviviruses in the near full-length polyprotein sequence suggests that the identified virus is a novel flavivirus that we have tentatively named Cuacua virus (CuCuV) based on geographical location (village name); its sequence has been deposited in GenBank (KX245154). Other flaviviral sequences were also detected in the *Mansonia* spp. mosquitoes, these sequences showed closest relationship to PCV (11 contigs mapping to several PCV genes/proteins (E, M, NS3 and NS5) with an amino acid identity of 46–87%; the contigs were 310–880 nt in length), to Nienokoue virus (2 contigs mapping to NS1 and NS5 with an 45–57% amino acid identity; the contigs were 1057 and 848 in length) as well as to Culex flaviviruses and other mosquito flaviviruses. No sequences related to known pathogenic flaviviruses were identified in the data sets.

### Rhabdoviruses *(Rhabdoviridae* family)

**Fig 3:**
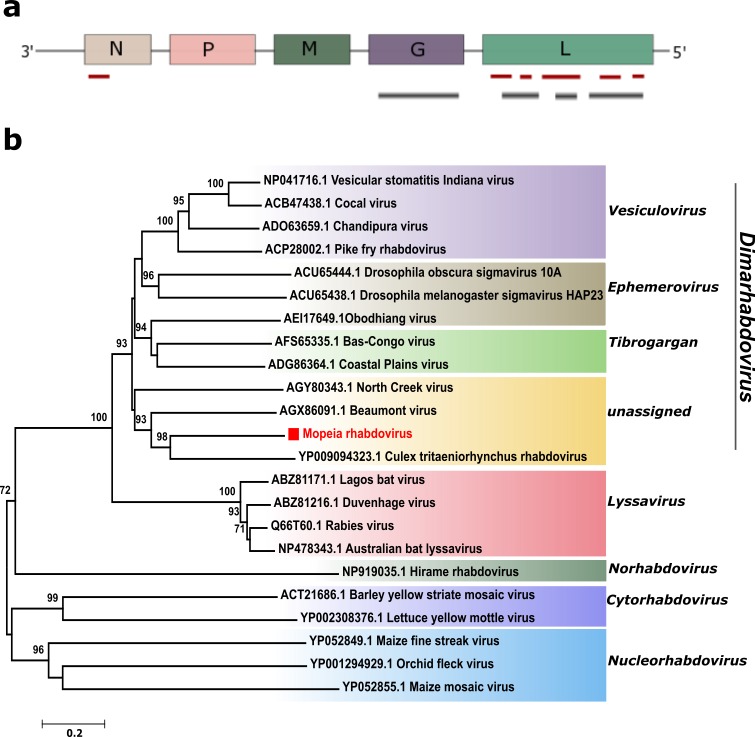
Schematic representation of the identified rhabdoviral genome from *Culex* (red bars) and *Mansonia* (black bars) (a). Phylogenetic analysis of the Mopeia rhabdovirus (MoPRV) detected in *Mansonia* spp. was performed based on a 512 amino acid sequence of the L segment (b).

Both the *Culex* and *Mansonia* spp. contained sequences related to rhabdoviruses, with 1% (36 reads) and 9% (89 reads) of all the viral reads in the respective data sets. In *Culex* spp., viral sequences were detected most closely related to *Culex tritaeniorhynchus rhabdovirus* (CTRV) (GenBank NC025384), with a sequence identity of 44–56% to the L segment (the RNA-dependent RNA polymerase gene). CTRV is an unclassified rhabdovirus identified in *Culex tritaeniorhynchus* mosquitoes from Japan in 2011 [6]. The assembled rhabdoviral reads generated 7 contigs (246–929 nt in length), which correspond to 50% of the L protein segment (6 contigs) and to a short region of the N segment (Nucleoprotein) (red bars in Fig 3A). The majority of the sequences in *Mansonia* spp. showed closest relationship to CTRV. As for the *Culex* rhabdoviral sequences, the reads showed low sequence identity (40–50%) at the amino acid level with the L and G segments of CTRV. The reads were assembled to obtain longer viral sequences, and this generated 6 contigs that covered 95% of the CTRV G segment (Glycoprotein gene) and 60% of the L segment, represented as black bars in Fig 3A. The identified rhabdoviral sequence exhibits high genomic diversity to known rhabdoviruses available, indicating a novel rhabdovirus that is tentatively named Mopeia rhabdovirus (MoPRV), based on geographical location (district name); the sequence has been deposited in GenBank (KX245155). Phylogenetic analysis of the MoPRV partial L protein sequence recovered from *Mansonia* spp. clustered together with CTRV in an unassigned group (Fig 3B). The rhabdovirus sequences with homology to CTRV, found in both the mosquito genera, show a nucleotide identity of 74–76% and an amino acid identity of 90–96% to each other. This suggests that the detected rhabdoviral sequences in both the genera are from the same virus or at least from two closely related viruses. Other rhabdoviral sequences related to Beaumont virus, North creek virus and Perinet virus were also identified, in *Mansonia* spp. mosquitoes, showing identity (34–78%) to the polymerase protein gene.

### Iflaviruses *(Iflaviridae* family)

A large number of sequences (2870) related to the *Iflaviridae* family were identified in *Culex* mosquitoes. These sequences were most closely related (24–63% amino acid similarity) to Nasonia vitripennis virus (GenBank FJ790487), Brevicoryne brassica picorna-like virus (GenBank NC009530, AKJ70949.1), Lygus lineolaris virus 1 (GenBank AEL30247.1) and Sacbrood viruses (GenBank AJA38041.1, AKG24959.1). No similarities were observed for *Iflaviruses* at nucleotide level.

### Other viruses

In addition to flaviviruses, rhabdoviruses and iflaviruses, other viral families were also detected in the two mosquito species (Fig 1A and 1B). In *Culex* spp., reads related to *Dicistroviridae*, *Picornaviridae*, *Parvoviridae* and unclassified ssRNA viruses were detected. A large number of sequences related to unclassified negative-strand ssRNA viruses and dsRNA viruses were identified in *Mansonia* spp.

## Discussion

In the present study, viral metagenomics was used to identify mosquito-associated viruses in the Zambezia province of Mozambique. This is the first study where high-throughput sequencing has been used to explore the viral communities of mosquito populations in Mozambique. In the two mosquito genera (*Culex* and *Mansonia*), a large number of viral sequences were identified, some of which had close or distant relationships to viral families such as *Flaviviridae*, *Rhabdoviridae* and *Iflaviridae* etc. while others appeared to be associated with unclassified viruses.

Viruses within the genus *Flavivirus* can be divided into three groups based on their arthropod and vertebrate host associations [20, 21]. Dual-host viruses that are capable to transmit between vertebrates and arthropods (mainly mosquitoes and ticks), vertebrate-only viruses, also known as no-known-vector viruses (NKVs) [22] which can be transmitted between vertebrates without any vector, and insect-specific flaviviruses (ISFs) which only transmit between arthropod vectors [23]. The novel flavivirus (Cuacua virus), genetically characterized in this study from the *Mansonia* spp. mosquitoes, was most closely related to NAKV and PCV and clusters with the insect-only group. A unique feature of all known ISFs are that they possess a slippery heptanucleotide sequence to express a novel overlapping gene processed by -1 PRF in the NS2A-NS2B region; the presence of this site in the CuCuV genome further supports that it belongs to the ISF group [19]. Insect-specific flaviviruses have been discovered in different mosquito populations globally. Cell fusing agent virus (CFAV) was the first isolated ISF from *Aedes aegypti* cell cultures in 1975, in Puert Rico [24]. Kamiti River virus was isolated from *Aedes* spp in Kenya [25], and the first CxFV was detected in *Culex* spp. from Japan [26]. With the advent of new molecular tools and growing interest in the mosquito virome, several new ISFs have been reported. Aedes flavivirus, Hanko virus, Culex theileri flavivirus, Quang Binh virus and Mercadeo virus are just a few of the newly discovered ISFs in last few years [27–32]. ISFs are believed to be maintained in the mosquito population through vertical transmission, although horizontal transmission between mosquitoes can also occur for some viruses [33, 34]. Different studies have shown that ISFs replicate in mosquito cells *in vitro* and *in vivo* but cannot infect or replicate in the vertebrate cells lines that have been tested [33, 35–37]. Phylogenetic analysis of these viruses has, as mentioned, placed them in a separate branch within the *Flaviviridae* family [38].

Rhabdoviruses are present in a wide range of hosts, including different plants, vertebrates and insects. Currently, there are 17 recognized and proposed genera present in the family, and many rhabdoviruses await taxonomic classification [39]. Because of the high genetic diversity of the viruses from different hosts, a large number of viruses have been placed in an unassigned group within the family. Previous studies have shown that arthropod vectors play a role in rhabdovirus transmission through horizontal transmission [40]. In the current study, several sequences related to the *Rhabdoviridae* family were identified in both the mosquito genera, and these showed most similarity to CTRV, with an amino acid identity of 40–65% (G, N and L segments). The detected novel rhabdovirus sequence also share similarity with Beaumont virus (43% at amino acid level) which was previously isolated in Australian mosquitoes [41]. Phylogenetic analysis of the partial L ORF showed that the identified rhabdovirus (Mopeia rhabdovirus) was assigned to an unclassified group within the dimarhabdovirus group and clustered with CTRV. In Mozambique, Mossuril virus is the only rhabdovirus that has been isolated from Culex mosquitoes [42], however, there is no similarities between Mossuril rhabdovirus and MoPRV.

The current findings are in agreement with the previous studies and demonstrate that mosquitoes harbor a rich and diverse virome [2, 3, 41]. Although we have focused on genetically characterizing only two of the viruses discovered in this study, the data show that a large number of undiscovered insect-specific flaviviruses, rhabdoviruses and a diverse range of unclassified viruses exist in the mosquito populations in Mozambique. The evolution of ISVs is not well described as little is known about their host range, ecology and distribution, which have to be further investigated. Recent studies suggest that many ISVs [39, 43] have probably evolved and diversified with their insect hosts over a very long period of time [3]. There has been no evidence that insect-specific viruses are pathogenic and cause diseases in animals or humans. However, it appears that numerous existing arboviruses may have evolved from being ISVs to dual-host viruses [3]. Certain ISVs can also potentially influence the vector competence for different pathogenic arboviruses as it has been shown that mosquitoes infected with certain ISFs are less susceptible to secondary infections, and reduced arboviral replication is sometimes observed in co-infected mosquito cell lines [5, 36, 37, 44]. With additional studies on co-infection dynamics in mosquitoes, ISVs may be used as biological control agents for mosquito-borne diseases. Therefor, further analysis of the microbial diversity in arthropod vectors will increase the understanding of viral evolution as well as could potentially contribute to control aspects of pathogenic arboviruses.

## Supporting Information

S1 Table List of primers used in the study. (DOCX)

S2 Table Number of reads related to each viral family classified from *Culex* and *Mansonia* species. (DOCX)
