# Supplementary material for: Discovery of Novel Viruses in Mosquitoes from the Zambezi Valley of Mozambique
Source: PLoS One. 2016 Sep 28;11(9):e0162751. doi: 10.1371/journal.pone.0162751 (PMC5040392; doi:10.1371/journal.pone.0162751)
Supplement: S1 Table — (DOCX) [file pone.0162751.s001.docx]

| Cuacua virus primers | |
| --- | --- |
| 1b_F | TAC CAA AGG ATG CGT GTC C |
| 1b_R | GAA TAG GTA GCA TTA ACA TCA CA |
| 2_F | GTG ATG TTA ATG CTA CCT ATT C |
| 2_R | TTGA AGG ATC CAA CAA AGT CAT |
| 3_F | TTT GGA GTT GCA GGG GTG G |
| 3_R | AGA TGT TAG TCT TCG TCC ATG |
| 4_F | AAG ATG AAA AGC TGT TTA TGC C |
| 4_R | TCT CCA TCC ACG TCG CTG T |
| 5_F | ATG GGC AAG TGG CTT GAG A |
| 5_R | TTT CCA TCT GCC ACG TGC TT |
| 6_F | ACT CGC GTT GTT CTG GTT GA |
| 6_R | TTT GTG CTT GCT AGC GTG TG |
| 7_F | TTGG CTT TCC TGG ACC TGG |
| 7_R | GTG GCC ACG AAA CTG CAA G |
| 8_F | GGT GAC GTC AAT CCC TTG G |
| 8_R | CGG AGG ACC TCC AAA TGG T |
| 9_F | AGA AAT GGT TGA CCA TAA ATC C |
| 9_R | TCG TCG GCT ATC ATC CAC TT |
| Mopeia rhabdovirus primers | |
| Rhabdo_1F | TAA GAG CAA GTA CAG GGG TTG G |
| Rhabdo_1R | CTC AGC ACG TCC GTT GGA C |
| Rhabdo_5F | GCT GCA ATC ACG ACC CAC |
| Rhabdo_5R | CTT GAA GGA AGC TGT CGG G |
| Rhabdo_6F | GGA GCT GAC AGA GGA GTT CCT AG |
| Rhabdo_6R | CAG TCC GGA TAT TGT AGG GTC TC |
| Rhabdo_7F | AAC GTT CTG AGC ATA GGC CTG |
| Rhabdo_7R | AGA TGT GAA CGA GCC CGA C |
