# Supplementary material for: Discovery of Novel Viruses in Mosquitoes from the Zambezi Valley of Mozambique
Source: PLoS One. 2016 Sep 28;11(9):e0162751. doi: 10.1371/journal.pone.0162751 (PMC5040392; doi:10.1371/journal.pone.0162751)
Supplement: S2 Table — (DOCX) [file pone.0162751.s002.docx]

| Viral Family | Number of reads | |
| --- | --- | --- |
|  | *Culex* spp. | *Mansonia* spp. |
| Baculoviridae | 2 | - |
| Bunyaviridae | - | 10 |
| Chrysoviridae | 22 | - |
| Coronaviridae | - | 4 |
| Dicistroviridae | 156 | - |
| dsRNA virus environmental sample | - | 256 |
| Endornaviridae | 1 | - |
| Flaviviridae | 19 | 257 |
| Iflaviridae | 2870 | 2 |
| Orthomyxoviridae | - | 10 |
| Parvoviridae | 46 | - |
| Phycodnaviridae | - | 1 |
| Picornaviridae | 42 |  |
| Polydnaviridae | 1 | - |
| Reaoviridae | - | 7 |
| Retroviridae | - | 1 |
| Rhabdoviride | 36 | 89 |
| ssRNA negative strand virus | - | 3 |
| ssRNA positive strand virus | - | 6 |
| Unclassified ssRNA negative strand  viruses | - | 327 |
| Unclassified ssRNA positive strand  viruses | 45 | 3 |
| Unclassified viruses | - | 7 |
| Uncultured virus hypothetical protein | 26 | - |
| Virigaviridae | 1 | - |
| **Total** | **3269** | **983** |
